# Policy: Healthier Housing Ahead

**Published:** 2005-05

**Authors:** Katrina Smith Korfmacher

Community-based organizations around the country are working to promote healthy and affordable housing for all. Yet reduced government funding and increased competition for foundation support make it even harder to redress housing-based health disparities. In early March 2005, the Alliance for Healthy Homes convened nearly 50 leaders from community groups around the country to plan how to work together to build a national movement for healthy homes in the face of political and funding challenges.

Several groups shared recent local successes and strategies. Cleveland has passed a city ordinance that provides incentives for landlords to make housing lead-safe. In San Diego, the Environmental Health Coalition trained *promotoras* (community health promoters) to teach families and code inspectors about home environmental hazards; the coalition is also organizing citizens in support of a proposed lead-safe housing ordinance. The Boston Urban Asthma Coalition reported progress in promoting good air quality in schools, getting insurers to pay for asthma patient education, and referring asthma patients to city building inspectors for targeted housing code enforcement. These and other experiences provided fodder for workshops on enhancing code enforcement, strengthening ordinances, working with the media to communicate healthy homes issues, and funding housing improvements.

The Alliance for Healthy Homes presented a plan for combining forces with the National Center for Healthy Housing to integrate research, policy, and practice into practical solutions for communities. Community leaders also stressed the need for a separate “uncensored voice” to speak on behalf of low-income communities that suffer most severely from unhealthy housing. The advocates in attendance agreed to pursue forming a new network to serve as this voice for the people.

The meeting highlighted the need to build recognition of the importance of healthy housing and the political will to ensure it through new partnerships at many levels. Community groups can organize residents and promote local policy changes. Local and national groups concerned with education, health, criminal justice, economic development, and poverty can include healthy housing among their central objectives. Researchers can focus further attention on the health risks posed by substandard housing, test and evaluate practical solutions, and educate policy makers and the media.

At the federal policy level, the Alliance for Healthy Homes and the National Center for Healthy Housing are drafting federal healthy homes legislation to comprehensively address the current policy gaps. This legislation could provide the rallying point for community advocates, special interest groups, researchers, and others to move healthy and affordable housing to the central position it deserves on our national agenda.

